# Assessing quit interest and the correlates and preferred ways of quitting snus in Norway: A cross-sectional study

**DOI:** 10.18332/tid/209194

**Published:** 2025-10-09

**Authors:** Gunnar Sæbø, Ingeborg Lund

**Affiliations:** 1 Department of Alcohol, Tobacco and Drugs, Norwegian Institute of Public Health, Oslo, Norway; 2 Group for Public Health Statistics, Norwegian Directorate of Health, Oslo, Norway

**Keywords:** snus cessation, nicotine addiction, quit aids, smokeless tobacco, tobacco endgame

## Abstract

**INTRODUCTION:**

Snus is currently the most used tobacco product in Norway. This study aims to identify the overall cessation interest among Norwegian snus users, the preferred quitting aids, and correlates of cessation interest.

**METHODS:**

Survey data were collected by the Norwegian Directorate of Health through a nationwide web panel, with respondents completing an online questionnaire. Three time points (two in 2018, one in 2019) were pooled, resulting in n=820 snus users. Descriptive statistics and adjusted multinomial logistic regression were applied to identify the extent of snus cessation behavior and factors associated with overall cessation interest.

**RESULTS:**

In all, 58.5% had attempted to quit snus, while 52.7% expressed current plans to quit. Of all snus users, 26.6% had never tried to quit and did not intend to quit in the future; 35.7% had either previously tried to quit but held no current quit plans, or they had never attempted to quit but were willing to try in the future. The remaining 37.7% had both tried to quit and intended to try again. Frequently preferred quitting aids were: quit on my own, mobile app, and nicotine-free snus. Higher interest in quitting was associated with younger age (AOR=0.94, p<0.001), living in western (AOR=2.27, p=0.019) or northern (AOR=2.60, p=0.022) Norway, perceiving snus use as hazardous to health (AOR=2.37, p<0.001), using snus daily (AOR=2.83, p<0.001), and non-smoking (AOR=0.53, p=0.033). Cessation behavior was not statistically associated with education level or income, after controlling for covariates.

**CONCLUSIONS:**

The majority of snus users are interested in quitting, especially those who are young and worry about their own health. We found no evidence of a social gradient in cessation interest.

## INTRODUCTION

Even if Swedish snus is prohibited in most countries, including the European Union (EU), this smokeless tobacco product is still allowed on the market in Sweden and Norway^1^. In Norway, snus is now the most frequently used tobacco product^2^, with a prevalence of 19% current use in the adult population (16–74 years) and 29% current use among youth aged 16–25 years^3^. Despite being considerably less harmful than cigarettes^4,5^, snus use may still be addictive and hazardous to health^6^, and there are currently public health concerns related to both initiation of snus use among adolescents^6^ and maintained nicotine addiction among former smokers who have used snus to quit cigarette smoking^7^. Lack of willingness to quit nicotine products altogether also weakens the health promoting push for a tobacco ‘endgame’ goal in tobacco preventive initiatives^8^.

However, little is known about the spread of quit interest, quit plans and preferred strategies to quit among current snus users in Scandinavia. Previous international studies of snus cessation are mainly evaluations of participation in cessation programs or interventions, either from the US or Southern Asia^9-11^. These studies have been conducted among users motivated, or at least with an incentive, to quit; they also address different and more hazardous smokeless products than low-moisture Swedish snus, such as dipping or chewing tobacco^12^. Another research thread has explored snus as a way of quitting cigarette smoking; a partially controversial topic^13,14^ that has been the subject of a systematic review and meta-analysis, suggesting some weak evidence of use of snus for smoking cessation^15^.

When snus becomes the dominant tobacco user product, like in Sweden^16^ and Norway, it also becomes relevant for tobacco and public health research to address snus cessation with similar approaches and concepts as in smoking cessation research. For instance, there is evidence to suggest that smokers move through ‘stages of change’ before quitting^17^ and that medical nicotine replacement therapies may work as a cessation aid in this process^18^. Yet, many smokers also quit on their own^19^. Smokers with long education have been quitting to a larger extent than smokers with short education, resulting in a strong social gradient among remaining smokers^20^. We do not know if similar dynamics play out among snus users who contemplate cessation or try to quit.

Hitherto, few studies have addressed interests and motivations to quit and preferred cessation methods/strategies among snus users. Apart from the study of Sohlberg and Wennberg^14^, we have not found any study from Norway or Sweden that addresses cessation or quitting of Swedish snus as an independent and major research problem. With this background, the purpose of this article is to identify: 1) overall quit interest among current Norwegian snus users by assessing the extent of previous quit attempts and future quit plans, 2) preferred quitting aids, and 3) significant correlates of snus cessation behavior. With regard to the last, we specifically aim to explore whether the known drivers of cigarette smoking cessation also apply to snus cessation – particularly, whether quit interest is higher among younger individuals, those with higher level of education, and those who perceive greater health risks^21^.

## METHODS

### Data

We make secondary use of data collected on behalf of the Norwegian Directorate of Health as part of the monitoring of national tobacco behavior before and after the Stoptober campaign^22^ to explore our own original research questions. The Stoptober media campaign, known from several countries around the world, motivates smokers and snus users to make a quit attempt during the month of October and stay tobacco-free for the following 28 days. Stoptober ran annually in Norway from 2018 to 2022, and pre and post campaign data of national tobacco use were collected in web surveys each of these years.

### Recruitment of participants

An invitation to participate in the monitoring study of Stoptober was sent by email to people who had pre-registered as smokers or snus users in a national consumer web panel consisting of about 85000 people, administered by the independent data collector Norstat. The sample was randomly selected from this panel, based on stratification of age, gender and region, to ensure proportional representation. When incoming responses reached a pre-set number (500 tobacco users), data collection was stopped. To exclude the same individual from participating two years in a row, respondents were excluded. To accommodate participation in the surveys, respondents received points that could be accumulated and, when reaching a certain number, be exchanged for a prize.

### Analytical sample

Participants were aged ≥18 years and had preregistered in the panel as tobacco users. They completed an online questionnaire covering tobacco use, previous quit attempts, future interest in quitting, knowledge of quitting methods, preferred quitting aids, and the presence or absence of a supportive social network. All tobacco-related questions in the study were mandatory, which minimized the amount of missing data.

While the Stoptober campaign primarily addresses smoking behavior, data were also collected on snus use at the first three time-points of the time series, although only among occasional smokers. Data from these three time-points (pre and post campaign in 2018, pre campaign in 2019) were pooled for the present analysis. This resulted in n=820 snus users (>18 years). It is important to note that this dataset does not consist exclusively of snus users who were directly exposed to the campaign. Rather, the sample of snus users was collected independently of whether they had noticed the campaign or not.

### Ethics

The data collector Norstat complies with the general data protection requirements in GDPR as well as the codes and guidelines developed by the European Society for Opinion and Marketing Research (ESOMAR). Informed consent was obtained from all participants. The data received for this analysis were anonymized and contained non-sensitive information only, and the survey was thus exempt from further ethical approval.

### Measures

The structured online questionnaire used to collect the data was specifically designed to monitor tobacco use at the national level before and after the Stoptober campaign. The variables selected from the full data set for use in this study are detailed below.

*Outcome variables*

Tobacco use

This was measured by the following questions: ‘Do you use snus?’ and ‘Do you smoke (cigarettes, roll-your-own, pipe, cigars/cigarillos)?’. Response categories were: ‘yes, daily’ (for snus use only); ‘yes, occasionally’, and ‘no’.

Snus cessation behavior

This was measured by asking: ‘Have you tried to stop using snus?’. The response categories were: yes, once; yes, 2–3 times; yes, 4–5 times; yes, ≥6 times; and ‘no, I have never tried, and I do not remember/do not want to answer’. And then ‘Are you planning to try to quit using snus?’, with response categories: ‘I am considering quitting within the next month’, ‘I am considering quitting within 1–3 months’, ‘I am considering quitting within 4–6 months’, ‘I am considering quitting but not in the first 6 months’, ‘I am not considering quitting snus’, and ‘Do not know’. These two variables were first assessed independently, then combined into one variable intended to measure the overall interest in quitting, with ‘no’ quit interest (no quit attempt or cessation plans, including do not know and do not want to answer responses, to simplify the presentation) coded as 1, ‘moderate’ interest (either having tried to quit only or holding plans to quit only) as 2, and ‘high’ interest (both having conducted quit attempts and holding quit plans) as 3. Although the resulting outcome variable may be considered a somewhat crude measure of cessation interest, we argue that combining past behavior with future motivational intentions creates a novel two-dimensional index of quit interest. This approach simultaneously captures an underlying measure of the intensity of cessation interest.

Respondents who had previously attempted to quit were also asked to tick for quitting aids used at last quit attempt, and those who held current plans were asked about preferred quitting aids to be used in the planned quitting of snus. The list of quitting aids included: nicotine remedies (gum, patches etc.), prescribed medicine (such as champix or zyban), quit line (‘Slutta’), quit course, digital sites dedicated to cessation, the mobile app ‘Slutta’(‘Quitting’, a smartphone-based smoking cessation intervention offering daily motivational messages and advice, drifted by the Norwegian Directorate of Health), health or medical personnel, nicotine-free snus, self-help books/brochures, alternative methods (hypnosis, acupuncture etc.), electronic cigarettes, other aids, and finally, quit on my own. For the present analysis, quitting aids were recoded and merged into five groups: nicotine remedies, mobile app ‘Slutta’, nicotine-free snus, others, and quit on my own.

*Covariates*

Demographics and socio-economic status were controlled for as potential confounders by means of gender (male, female), age (continuous), geographical region (Oslo, southern, east, west, mid, north), education level (primary/secondary, university ≤3 years, university ≥4 years), and personal income in NOK (<200.000; 200.000–399.000; 400.000–599.000; 600.000–799.000; 800.000–999.000; ≥1 million; and no info) (Table 1).

**Table 1:** Participant characteristics (N=820)

*Characteristics*	*%*	*n*
**Year**		
2018 – pre campaign	36.8	302
2018 – post campaign	38.8	318
2019 – pre campaign	24.4	200
**Gender**		
Male	62.7	514
Female	37.3	306
**Age** (years)		
18–29	30.6	251
30–49	44.6	366
≥50	24.8	203
Mean ± SD	39.03 ± 13.52	
**Geographical region**		
Oslo (capital city)	18.9	155
South	6.6	54
East	30.7	252
West	18.9	155
Mid	16.0	131
North	8.9	73
**Education level**		
Primary/Secondary	36.2	297
University ≤3 years	29.8	244
University ≥4 years	32.7	268
Other*	0.6	5
No information*	0.7	6
**Personal income** (NOK)		
<200.000	10.1	83
200.000–399.000	14.4	118
400.000–599.000	34.8	285
600.000–799.000	17.7	145
≥1 million	6.5	53
Do not want to answer/Don’t know	9.5	78
No information*	0.7	6
**Risk perception (‘I worry about how snus affects my health’)**		
1=Totally disagree	23.2	190
2	26.0	213
3	24.1	198
4	15.6	128
5=Totally agree	10.0	82
6=Do not know	1.1	9
Mean ± SD (after recoding 6 into 3)	2.64 ± 1.27	
**Do you smoke?**		
No	84.3	691
Occasionally	15.7	129
**Do you use snus?**		
Occasionally	13.4	110
Daily	86.6	710
**Have you tried to quit snus?**		
No	39.9	327
Once	17.0	139
2–3 times	26.5	217
4–5 times	8.3	68
≥6 times	6.7	55
Don’t remember/don’t want to answer	1.7	14
**Are you planning to try to quit snus?**		
No	32.9	270
Yes, but not in the first 6 months	22.6	185
In 4–6 months	8.4	69
In 1–3 months	11.8	97
In the coming month	9.9	81
Don’t know	14.4	118

* Defined as missing values in regression analyses. NOK: 1000 Norwegian Kroner about US$100.

Risk perception of snus use was measured by agreeing or disagreeing with the following statement ‘I worry about how snus affects my health’ on a 5-point Likert scale (1 = ‘totally disagree’ to 5 = ‘totally agree’). Since the respondents were not provided with qualitative descriptions for the middle categories (2–4), the variable was treated as continuous. Do not know responses were recoded into the middle category (=3).

### Statistical analysis

Descriptive statistics was used to identify the extent of snus cessation behavior. Bivariate associations were assessed using chi-squared tests, Cramer’s V (for nominal variables, such as quitting aids used previously and in the planned future) and Spearman’s rho (for ordinal variables). All tests were two-tailed.

Adjusted multinomial logistic regression was applied to identify associations between demographics, socio-economic status, risk perception of the health hazard of snus, tobacco use status, and overall snus cessation interest. In this model, age and risk perception of health hazard of snus use were entered as continuous ‘covariates’, all other controls were entered as categorical ‘factors’.

The likelihood ratio chi-squared test indicates that the full regression model (Table 4) is a significant improvement in fit over the null model [χ^2^(36)=238.401, p<0.001]. Also, the results of the Pearson [χ^2^(1552)=1589.587, p=0.248] and Deviance [χ^2^(1552)=1497.288, p=0.837] chi-squared tests are both non-significant, which indicates that the model fits the data well.

Sensitivity analyses were conducted to assess whether replacing our education variable with a weighted version based on national statistics would yield significantly different regression results.

No weighting or adjustment for strata was applied. Analyses were conducted using SPSS v30.

## RESULTS

### Descriptives

The sample consisted of 62.7% males and 37.3% females, with a mean age 39.03 ± 13.52 years (Table 1); 58.5% had previously attempted to quit snus, either once or more times, while 52.7% expressed current plans to quit, either in the coming six months or in the longer term (more than six months) (Table 1). While only 1.7% did not remember or did not want to answer the question about previous quit attempts, 14.4% was unsure whether they were planning to try to quit snus or not in the future. Of those who had previously tried to quit, 71.0% had tried to quit more than once (not shown in table).

There were missing values for 16 individuals who lacked information on either education level or personal income, or both. About half of the snus users (49.2%) did not worry about the health hazards of their snus use (ticking for 1 or 2 on the risk scale). About 1 in 4 (25.6%) agreed that they worried about how snus affected their health (ticking 4 or 5 on the risk scale).

**Figure 1:**
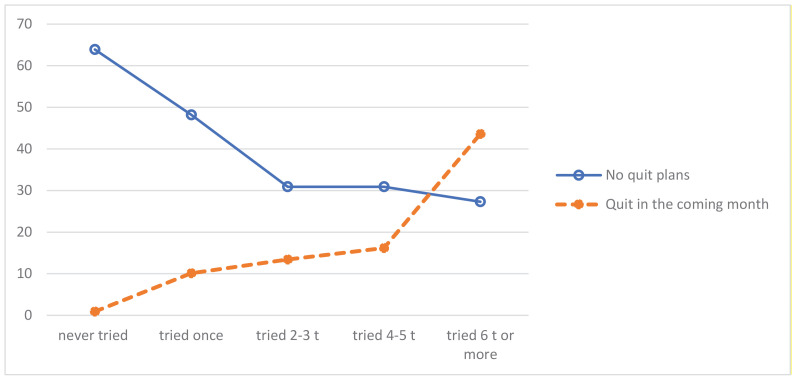
Future quit plans (%) by number of previous quit attempts (N=820)

The two cessation variables were strongly correlated (Spearman’s rho = 0.42, p<0.001) (Table 2). The more quit attempts snus users had made, the more likely they were to express quit plans in the near future. Figure 1 illustrates this relationship by comparing the distribution of the extreme categories of the ‘quit plans’ variable based on the number of previous quit attempts.

**Table 2:** Future quit plans (%) by number of cessation attempts

	*Number of cessation attempts*					
	*Never tried or don’t remember*	*1*	*2–3*	*4–5*	*≥6*	*Total*
No plans or don’t know	63.9	48.2	30.9	30.9	27.3	47.3
Quit > 6 months ago	23.8	24.5	24.4	16.2	10.9	22.6
Quit 4–6 months ago	7	8.6	12.4	4.4	5.5	8.4
Quit 1–3 months ago	4.4	8.6	18.9	32.4	12.7	11.8
Quit the coming month	0.9	10.1	13.4	16.2	43.6	9.9
Total, n	341	139	217	68	55	820

χ^2^=200.339; significance ≤0.001. Spearman’s ρ=0.42; significance ≤0.001.

### Overall quit interest

In all, 26.6% had never tried to quit and did not intend to quit in the future, consequently expressing no interest in quitting and 35.7% had either previously tried to quit but held no current plans of quitting, or they had never tried to quit, but expressed willingness to try in the future, thereby holding a moderate interest to quit (Table 3). Finally, 37.7% of the snus users had previously tried to quit and aimed to try again in the future, thereby expressing a high interest to quit. The response pattern of the utilized snus cessation variable remained relatively stable over time (Table 3), with no significant differences across the three time points.

**Table 3:** Overall interest in quitting snus (%) by sub-sample

*Overall interest in quitting snus*	*2018-pre*	*2018-post*	*2019-pre*	*Total*
No quit attempt or plans to quit (no interest)	26.8	26.7	26.0	26.6
Quit attempt only or quit plans only (moderate interest)	32.8	37.1	38.0	35.7
Both quit attempt and quit plans (high interest)	40.4	36.2	36.0	37.7
Total, n	302	318	200	820

χ^2^=6.162; significance=0.706.

### Quit aids

The most frequently mentioned aids/methods applied in the last unsuccessful quit attempt were: quit on my own (49.9%), the mobile app ‘Slutta’ (27.3%), and nicotine-free snus (25.9%) (Table 4). Willingness to use an aid in a future quit attempt is generally higher though, and the percentage who prefers to quit on their own next time decreases to 37.2%. Snus users who had previously tried to quit and who were willing to try again in the future, tended to prefer using the same aid of quitting.

**Table 4:** Aids used at last quit attempt, aids most likely to be used in the next quit attempt, and statistical association between using the same aid in next quit attempt as in the last quit attempt, multiple choices

*Aids*	*Last quit attempt (%)*	*Next quit attempt (%)*	*% (of N who used aid last time) planning to use same aid again*	*Cramer’s V*
Nicotine remedies (gum, patches etc.)	10.4	13.4	56.7 (30)	0.45
Mobile app (‘Slutta’)	27.3	33.5	70.2 (94)	0.49
Nicotine-free snus	25.9	36.5	75.0 (80)	0.47
Other aids*	9.8	16.9	40.0 (20)	0.18
Quit on my own	49.9	37.2	50.0 (128)	0.33
Total, n	479	403	290	

* Includes prescribed medicine (such as champix or zyban), quit line/quit course, digital sites dedicated to cessation, health or medical personnel, self-help books, brochures, electronic cigarettes, and alternative methods (hypnosis, acupuncture etc.)

### Regression analysis

Compared to having ‘no’ cessation interest, expressing a ‘high’ interest in quitting snus (having current quit plans and previous quit attempts) was positively associated with being younger (OR=0.95, p<0.001; AOR=0.94, p<0.001), living in the western (OR=1.98, p=0.019; AOR=2.27, p=0.019) or northern (OR=1.89, p=0.081); AOR=2.60, p=0.022) regions of Norway, perceiving snus use as hazardous to health (OR=2.23, p<0.001; AOR=2.37, p<0.001), using snus daily rather than occasionally (OR=2.38, p<0.001; AOR=2.83, p<0.001), and not being a smoker (OR=0.83, p=0.452; AOR=0.53, p=0.033) (Table 5). However, after multiple controls, a ‘high’ interest in quitting was not significantly associated with gender, education level, or personal income.

**Table 5:** Multinominal regression. Outcome variable: overall cessation interest; Reference: no cessation interest (N unadjusted ORs=820, N adjusted ORs=805)

*Variables*	*Overall cessation interest*											
	*Quit attempt or plans only (moderate)*						*Plans + quit attempt (high)*					
	*OR*	*95% CI*	*p*	*AOR*	*95% CI*	*p*	*OR*	*95% CI*	*p*	*AOR*	*95% CI*	*p*
**Gender**												
Male	0.88	0.61–1.25	0.491	1.12	0.73–1.71	0.619	0.66	0.46–0.94	0.023	1.24	0.79–1.95	0.351
Female ®	1			1			1			1		
**Age**	0.97	0.96–0.98	<0.001	0.97	0.95–0.98	<0.001	0.95	0.94–0.96	<0.001	0.94	0.92–0.95	<0.001
**Region**												
North	1.78	0.83–3.80	0.138	1.69	0.76–3.76	0.202	1.89	0.93–3.86	0.081	2.60	1.15–5.91	0.022
Mid	1.06	0.60–1.89	0.833	0.92	0.49–1.71	0.785	0.69	0.39–1.22	0.202	0.85	0.43–1.67	0.630
West	2.18	1.20–3.99	0.011	1.97	1.02–3.80	0.043	1.98	1.12–3.52	0.019	2.27	1.14–4.49	0.019
East	1.79	1.07–2.98	0.025	1.60	0.92–2.78	0.093	1.11	0.67–1.82	0.689	1.32	0.73–2.39	0.355
South	1.62	0.72–3.66	0.242	1.00	0.41–2.44	0.999	1.41	0.65–3.08	0.389	1.27	0.52–3.15	0.600
Oslo (capital) ®	1			1			1			1		
**Risk perception**	1.33	1.13–1.56	<0.001	1.40	1.18–1.66	<0.001	2.23	1.89–2062	<0.001	2.37	1.98–2.84	<0.001
**Smoking**												
Occasionally	1.22	0.76–1.96	0.402	0.87	0.52–1.46	0.601	0.83	0.51–1.35	0.452	0.53	0.30–0.95	0.033
No smoking ®	1			1			1			1		
**Snus use**												
Daily	2.32	1.42–3.80	<0.001	2.51	1.47–4.30	<0.001	2.38	1.46–3.88	<0.001	2.83	1.58–5.07	<0.001
Occasionally ®	1			1			1			1		

* Also controlling for education level and personal income. ® Reference categories.

Except for smoking, all these variables also predicted a ‘moderate’ interest in quitting (having made a quit attempt only or having quit plans only) compared to having ‘no’ cessation interest. Once again, gender, education level, and personal income showed no significant effect on quit interest. However, an additional multinomial regression analysis, which differentiated between those with moderate interest who had made a quit attempt only and those who held quit plans only, found that males were significantly more likely to have made a quit attempt only, while females were more likely – though only near-significantly – to hold quit plans only (Supplementary file Table 1). Sensitivity analyses assessing the impact of replacing the education variable with a version weighted according to national statistics revealed no significant differences in the results.

## DISCUSSION

A majority of the snus users expressed real interest to quit snus, either by having conducted at least one previous cessation attempt or by expressing plans to quit in the future. Only one out of 4 snus users had never tried to quit and did not hold any future cessation plans.

Compared with previous studies from the South Asian region, the willingness to quit snus in Norway can be characterized as high. A study from India noted that only 38% of its users had intentions to quit^23^, while in Bangladesh only 10% of the female users studied intended to quit^24^. Compared to South Asia (which alone accounts for more than three-quarters of consumption of smokeless tobacco worldwide^25^), the higher willingness to quit in Norway may have socio-cultural explanations and also reflect the fact that tobacco control and information work on the harms of smokeless tobacco has progressed further in Scandinavia than in South Asia.

The willingness to quit snus in Norway also seems higher than the willingness to stop smoking^26^. Similar findings exist from Sweden^27^. A potential contributing explanation for this might be that many current snus users are former smokers, who have used snus as an aid to stop cigarette smoking^14,15^. Many of these individuals may view their snus use as temporary, and a step on the way towards complete freedom from nicotine. Research has suggested that smoking cessation occurs through several ‘stages of change’^17^, and snus use may represent one such stage if it temporarily replaces smoking – similar to how nicotine replacement therapies (NRTs) have previously been found to function^18^. Tellingly, a Swedish investigation of snus use among former smokers found that those who experienced physical and psychological effects from snus continued to use snus, while those who did not tended to quit^14^.

The high proportion of former smokers among snus users in Norway^28^ may also help explain the absence of a social gradient in the interest in snus cessation observed in our study. While cessation from smoking has been more successful in higher social strata, snus users who used to smoke represent a self-elected group who have already embarked on the road to quitting^29^. Any underlying social gradient would therefore be considerably attenuated. Furthermore, a contributor to the lack of social gradient in snus quitting might be that the use of snus itself has been less associated with social inequality since its popularity started to increase in the population around the turn of the century^30^. Lastly, today, all segments of the Norwegian society are aware of the harmfulness of nicotine, and there are indications to suggest that the time span from initiation of a tobacco product to the onset of cessation attempts are shorter in recent cohorts than in earlier times^31^. This fits well with the current results, where not only younger age but also awareness of health hazards is markedly associated with cessation plans and activity. Snus use may be particularly harmful to pregnant women (or more precisely, to the unborn child)^6^, but as an extensive study of 2528 pregnant women in Norway and Sweden showed, most female users stopped consuming snus when they recognized pregnancy^32^.

Snus cessation interest is particularly high in the Northern and Western regions of Norway, both of which are marked by a ‘coastal culture’ and the presence of primary industries, especially fisheries and farming^30^. In Norway, snus use is more prevalent among farmers and fishers (as well as craftsmen and cleaners) of both genders^30^, which possibly may indicate a kind of ‘hidden’ social gradient between different cultures in different regions. However, it is not obvious why quit interest should be higher in areas with many snus users than in areas with fewer snus users.

In line with previous studies of aids used to quit smoking^33^, we found a willingness also among snus users to prefer using the same aid or method in a planned future cessation attempt as in earlier unsuccessful quit attempts. However, the majority of those who wanted to quit, preferred to do so on their own, again in line with previous findings suggesting this to be a more effective way to quit, not only smokeless tobacco^34^, but also cigarettes^19,31^.

In summary, the findings of this study suggest that established conceptualizations in smoking cessation research are also applicable to studies on snus cessation. The general lack of previous studies on snus cessation, noted in the introduction of this article, possibly signals low research interest in this topic, potentially because quitting snus hitherto have been considered of little significance for public health. Swedish snus is markedly less hazardous to health than combustible cigarettes^5,6^. Some public health representatives have therefore viewed it as a legitimate smoking cessation aid for inveterate smokers; this includes the FDA^35^ and the American Cancer Society^36^ in the US, and the Royal College of Physicians^37^ in the UK. However, snus is still a highly addictive substance with known health risks to users^6^. From the point of view of public health, it is therefore important to prevent snus use initiation and to continue to promote snus cessation in the future. This goal will also be in line with WHO’s stance in tobacco control and the increasing push for tobacco endgame in many countries.

### Limitations

This study has several limitations. Firstly, the design is cross-sectional, which means that causation cannot be inferred. Secondly, our secondary use of these data to identify quit interest and its correlates is subject to certain statistical limitations. The Stoptober campaign encourages quit attempts, and combining two pre-campaign datasets with one post-campaign dataset may consequently introduce analytical inconsistencies due to potential campaign influence on the post-campaign data. However, since the sample was collected independently of whether the snus users had noticed the campaign or not and the response pattern of the utilized snus cessation variable also remained relatively stable over time (Table 2), we chose to proceed with the pooled data, to maintain statistical power. Nevertheless, we have exercised caution when interpreting the statistical estimates derived from the pooled data.

Thirdly, although the data were randomly selected from a web panel, the requirement that respondents be pre-registered as tobacco users suggests that the dataset’s representativeness should be critically evaluated. Compared to official national statistics on snus users, the analytical sample was reasonably representative in terms of snus use status and sociodemographics, except for an overrepresentation of respondents with higher level of education^38^. This may have contributed to a certain overestimation of quit interest. However, snus use has been much less associated with social inequalities than smoking, and even if there is now a tendency for highly educated people to no longer initiate snus to the same extent as low or middle-educated people^38^, we should not automatically extrapolate a higher quit interest among those with longest education. Nor did the sensitivity analyses replacing our education variable with a weighted version based on national statistics yield significantly different regression results.

Another potential source of bias may stem from the exclusion of daily smokers from the dataset. Questions about snus use were not posed to daily smokers, as most dual users of cigarettes and snus smoke only occasionally^39^. Aside from these possible biases, there is no reason for the snus users in this sample to differ systematically from other snus users in terms of their interest in quitting, nor are we aware of any crucial residual confounder.

The study is based on self-reported data, which to some extent may be hampered by both retrospective distortions (when it comes to the historical accuracy of memory) and current social desirability. Therefore, to avoid drawing conclusions from possible misrepresentations, we have intentionally not delved into the finely grained nuances of the cessation variables, neither those who are based on past experiences nor those who express current ambitions of changing behavior in the future. Also, as snus use is not as associated with the same social marginalization, stigma, and shame as smoking^40^, it is likely that the current normative snus climate has not resulted in a marked social desirability bias in the data.

Finally, the geographical generalizability of the results may be somewhat limited, as snus is banned in many countries, including those in the EU and Australia. However, given the widespread use of smokeless tobacco in South Asia, the findings may still be relevant to health authorities in countries such as India and Bangladesh.

## CONCLUSIONS

The majority of snus users report willingness to quit, either by having conducted quit attempts and/or holding plans to quit. About 1 in 4 expresses no interest to quit. Overall quit interest is higher among the young, those who use snus daily and especially among those that worry about the health hazards of snus. The lack of significant associations with education level suggests that snus cessation patterns may differ from smoking cessation patterns which tend to have a strong social gradient^41^.

## Supplementary Material



## Data Availability

The data supporting this research are available from the authors on reasonable request.
