# Supplementary material for: Assessing quit interest and the correlates and preferred ways of quitting snus in Norway: A cross-sectional study
Source: Tob Induc Dis. 2025 Oct 9;23:10.18332/tid/209194. doi: 10.18332/tid/209194 (PMC12510316; doi:10.18332/tid/209194)
Supplement: Supplementary file 1 [file TID-23-152-s1.pdf]

Supplementary table1: Multinominal regression. Outcome variable: Overall cessation interest (4 categories version). Reference category: No cessation interest. Unadjusted (OR) and adjusted (AOR) odds ratios, 95% confidence interval (CI) and p-value (N unadjusted ORs= 820, N adjusted ORs=805).

	Overall cessation interest:																	
	**Quit attempt, no plans (*moderate1*)**						**Plans, no quit attempt (*moderate2*)**						**Plans + quit attempt (*high*)**					
**Variables:**	OR	95% CI	p-val	AOR*	95% CI	p-val	OR	95% CI	p-val	AOR*	95% CI	p-val	OR	95% CI	p-val	AOR*	95% CI	p-val
Gender																		
-Male	1.16	[.75-1.79]	.506	1.72	[1.04-2.85]	.035	.62	[.39-.97]	.038	.63	[.37-1.06]	.083	.66	[.46-.94]	.023	1.24	[.79-1.96]	.348
-Female (ref)	-	-	-	-	-	-	-	-	-	-	-	-	-	-	-	-	-	-
Age	.96	[.94-.97]	<.001	.95	[.93-.97]	<.001	.99	[.97-.1.01]	.210	.99	[.97-1.02]	.443	.95	[.94-.96]	<.001	.94	[.92-.95]	<.001
Region																		
-North	1.11	[.43-2.87]	.828	1.11	[.41-3.01]	.838	2.78	[1.13-6.80]	.025	2.49	[.97-6.38]	.058	1.89	[.93-3.86]	.081	2.59	[1.14-5.87]	.023
-Mid	1.14	[.59-2.21]	.691	1.00	[.49-2.06]	.997	.95	[.43-2.07]	.889	.83	[.36-1.91]	.661	.69	[.39-1.22]	.202	.85	[.43-1.68]	.645
-West	1.79	[.89-3.60]	.103	1.46	[.68-3.14]	.332	2.78	[1.32-5.85]	.007	2.84	[1.28-6.32]	.010	1.98	[1.12-3.52]	.019	2.26	[1.14-4.48]	.019
-East	1.77	[.98-3.17]	.057	1.52	[.81-2.88]	.194	1.82	[.94-3.54]	.077	1.74	[.86-3.52]	.123	1.11	[.67-1.82]	.689	1.33	[.74-2.40]	.348
-South	2.28	[.96-5.43]	.063	1.27	[.48-3.34]	.633	.64	[.16-2.51]	.523	.53	[.13-2.16]	.372	1.41	[.65-3.08]	.389	1.29	[.52-3.18]	.587
-Oslo (capital) (ref)	-	-	-	-	-	-	-	-	-	-	-	-	-	-	-	-	-	-
Risk perception	1.17	[.97-1.40]	.099	1.26	[1.03-1.53]	.024	1.57	[1.30-1.91]	<.001	1.59	[1.29-1.95]	<.001	2.24	[1.90-2.64]	<.001	2.36	[1.97-2.83]	<.001
Smoking																		
-Occasionally	1.30	[.77-2.21]	.325	.86	[.48-1.54]	.612	1.11	[.61-2.02]	.722	.91	[.48-.1.74]	.777	.83	[.51-1.35]	.452	.54	[.30-.95]	.034
-No smoking (ref)	-	-	-	-	-	-	-	-	-	-	-	-	-	-	-	-	-	-
Snus use																		
-Daily	2.47	[1.36-4.49]	.003	2.50	[1.31-4.76]	<.005	2.14	[1.13-4.07]	.020	2.57	[1.27-5.21]	.009	2.38	[1.46-3.88]	<.001	2.84	[1.59-5.09]	<.001
-Occasionally (ref)	-	-	-	-	-	-	-	-	-	-	-	-	-	-	-	-	-	-

**Also controlling for educational attainment and personal income*

© 2025 Sæbø G. and Lund I.
